# Beyond Words: Associations between the Maternal Emotional Environment and Children’s Internalizing and Externalizing Symptoms

**DOI:** 10.1038/s41598-026-47543-1

**Published:** 2026-04-13

**Authors:** Ellie Mae Dorrans, Antony Manstead, Stephanie H.M. van Goozen

**Affiliations:** 1 School of Psychology, Cardiff University, Cardiff, UK; 2 Department of Clinical Neurodevelopmental Studies, Leiden University, Leiden, the Netherlands; 3 Neurodevelopment Assessment Unit (NDAU), Centre for Human Developmental Science (CUCHDS), School of Psychology, 70 Park Place, Cardiff, CF10 3AT UK

**Keywords:** Expressed Emotion, Maternal Facial Expressivity, Maternal Mental Health, Internalizing Problems, Externalizing Problems, Risk factors, Psychology, Human behaviour

## Abstract

Mothers are rich sources of emotional information for children. Childhood emotional and behavioral problems have been associated with maternal anxiety, depression, Expressed Emotion (EE), and facial expressivity. However, no study has examined these factors together. The current study adopted a multimodal approach to assess how the maternal emotional environment relates to severity of internalizing and externalizing problems in children. The sample included children (*N* = 134; aged 4–7; 68.7% male) referred for assessment by teachers for exhibiting psychosocial problems at school. To assess psychopathology, parents completed the Hospital Anxiety and Depression Scale (HADS) and the Child Behavior Checklist (CBCL). EE was measured during the Five-Minute Speech Sample (FMSS) using the Expressed Emotion Coding System; facial expressivity was assessed using AFFDEX analysis during the FMSS. Maternal mental health, EE, facial expressivity, and child outcomes were significantly related. Regression analyses showed that fewer and less intense maternal facial anger expressions during negative comments were the best predictors of more severe child internalizing and externalizing problems, alongside EE. These findings highlight the role of the maternal expressed emotion over and above maternal mental health problems in children’s psychosocial problems and can inform the development of parenting and emotion-based interventions to tackle emerging mental health problems in children.

**Supplementary Information:**

The online version contains supplementary material available at 10.1038/s41598-026-47543-1.

Early childhood problem behaviors, such as internalizing (e.g., low mood, anxiousness, and social withdrawal) and externalizing problems (e.g., conduct problems, oppositional behaviors, and hyperactivity), are amongst the earliest indicators of long-term adjustment issues^1^. Within the UK, approximately one in five children present with symptoms of internalizing problems, externalizing problems, or both^2^. Recent NHS longitudinal data indicate that these difficulties are not only common, but also becoming increasingly evident, with almost 40% of children aged 6–16 experiencing a deterioration in their mental health between 2017 and 2021^3^. Given that these difficulties typically emerge in early childhood and frequently persist into adulthood^1^, identifying factors associated with the severity of early mental health problems is critical for informing timely intervention, when developmental processes are more malleable^4^. Emotion socialization research emphasizes the parent-child relationship as the primary context of a child’s emotional development^5–7^, with evidence suggesting that parents who appropriately model emotions and provide a warm emotional climate have children with fewer internalizing and externalizing problems^8,9^. However, relatively little is known about how different components of the maternal emotional climate, particularly maternal facial expressivity alongside verbal emotional communication, relate to the severity of children’s internalizing and externalizing problems. Addressing this gap, the present study examined the associations between maternal mental health, emotional expressivity – indexed both verbally and facially – and children’s internalizing and externalizing difficulties.

## The Role of Parental Facial Expressivity

Parents model emotion-related behaviors through the appropriate expression, management, and regulation of their emotions^6^. Modeling is recognized as a key mechanism in Social Learning Theory, whereby children imitate and acquire parental behaviors and attitudes and integrate them into their own emotional and behavioral profiles^10^. Facial expressivity, typically indexed by expressive facial behaviors, is seen as a core construct of emotion socialization and plays a significant role in shaping a child’s social and emotional development^6^. Children who grow up in expressive families, where parents exhibit varied and more intense facial expressions, learn how to understand and express emotions over time^11,12^, and have more opportunities to engage in adaptive emotion-related behaviors.

Exposure to predominately positive facial affect is associated with improved socioemotional functioning^13^ and fewer internalizing and externalizing problems in young children^14^. Chronic exposure to overly negative parental models of emotions, however, is thought to elicit distress in the child, and an internalization of negative attitudes and emotion-related behaviors. This can manifest as more adverse socioemotional outcomes and externalizing problems in young children^13,15,16^. A lack of parental expressiveness can also negatively impact a child’s socio-emotional development, because children receive insufficient cues for understanding and managing their own and others’ emotions^11,17^. Finally, inconsistent emotional cues (e.g., expressing negative facial affect during a positive verbal exchange) can result in maladaptive emotional teaching and a lack of emotional security for the child^18^, which can lead to adverse child outcomes^18,19^. Thus, parents whose facial behavior is inappropriate for the context provide an emotional environment in which the child is less likely to understand or appropriately regulate emotions.

## Facial Expressivity and Parental Mental Health

Parental mental health can influence facial expressivity because expressivity is shaped by the way that parents recognize, express and regulate their emotions^20^. Although they often co-occur^21^, maternal anxiety and depression are distinct psychopathologies and contribute differently to an individual’s profile of expressivity. Maternal depression is associated with more displays of negative affect and fewer displays of positive affect^22^. Research on the relation between parental anxiety and facial expressivity is sparser with most of the evidence coming from research on parents with comorbid anxiety and depression. As a result, conclusions are inconsistent, with some studies reporting that anxious mothers are less positive and more negative in their facial behavior^23^, and others reporting only less positive facial behavior^24^, or both more positive and more negative facial expressivity^25^.

Few studies have examined inconsistent or reduced parental expressions. However, parents who struggle with monitoring and regulating their own emotions, as observed in those with depression and/or anxiety^26^, may show greater inconsistency in emotional socialization behaviors^27^. This suggests that parental mental health might result in maladaptive modeling behaviors, although no research has yet examined the relationship between parental expressivity and severity of child emotional and behavioral problems in a high-risk sample of parents and children. In the present context, high-risk samples are defined as families in which children present with elevated emotional or behavioral difficulties and in which parents exhibit heightened levels of psychopathology. These family contexts provide an important and unique opportunity to examine emotion socialization processes, because parental psychopathology and increased parenting stress can result in maladaptive emotional communication and modeling^20–27^, which may reinforce atypical child development.

## The Assessment of Parental Facial Expressivity

Most studies assess parental facial expressivity using self-report measures^13,14^. Although such self-reports are moderately accurate for positive expressivity, they are less so for negative expressivity^28,29^. Self-reports rely on self-awareness and accurate recall and are subject to cultural views and cognitive biases, this is thought to be especially pertinent for individuals with symptoms of anxiety or depression^30^.

As an alternative, observational coding of facial affect during mother-child interactions has been used^8,15^. This method not only provides a more objective assessment of facial expressivity, but also enables a focus on how maternal facial behavior is perceived by the child. Halberstadt et al.^31^ discussed the limitations of assessing a parent’s facial expressivity when interacting with their child, suggesting that measures of expressivity that do not directly involve the child are ‘a somewhat purer measure of parental expressiveness’^15^, p. 476). Parents display more positive and less negative expressions in direct interactions with their child^15^ and measures of expressivity taken outside interactions with the child are therefore believed to be a more accurate representation of parental expressivity, and its impact on the emotional climate. Observation coding of faces and behaviors, however, can also be influenced by misinterpretation and biases. This is especially pertinent when it comes to coding of dynamic and/or ambiguous facial behaviors^32^. When a facial display is subtle, rapidly changing or ambiguous, a coder may rely on the content of the subject’s speech or wider behaviors to interpret and make sense of what is observed^33^, which could lead to missed or inaccurate coding.

An emerging alternative to observer coding schemes is the use of automated facial expression analysis (AFEA) to detect facial behaviors. Literature supports the use of AFEA, and it is generally seen as a valid and reliable tool for use in emotion-based research^34^. This is primarily because AFEA is thought to be an efficient solution to overcome the aforementioned limitations of traditional, observer-based coding of facial expressions^35^, providing an objective measurement of ambiguous or subtle expressions and frame-by-frame analysis reducing the likelihood that facial expressions will be missed^36^. One such method is Affectiva’s AFFDEX, as implemented in iMotions (www.imotions.com). AFFDEX coding systems were developed from Ekman and Friesen’s^37^ Facial Action Coding System (FACS) and use algorithms to detect facial movement in terms of facial action units (FAUs). This system provides a dimensional scoring of FAU activation, with higher scores reflecting more intense movement. Thus far, very few studies have used AFEA to examine how emotion related behaviors from the mother influence the child. One recent proof of concept study found that parents’ negative facial expressions were associated with higher levels of adolescents’ internalizing symptoms^38^. Our study is the first to use AFEA to assess associations between parental mental health, facial expressivity and psychopathology in young children.

## Expressed Emotion (EE)

Children do not experience parental emotional expression in isolation; parents provide a network of verbal and nonverbal emotional communications that make up the child’s emotional environment^6,7^. Including verbal expressions can further our understanding of how emotional and behavioral problems develop and persist in children. The construct of ‘Expressed Emotion’ (EE) reflects the positive or negative attributions that parents make about their child^39^ and the quality of affective interactions in a parent-child dyad (i.e., levels of negativity or warmth). High levels of EE (i.e., high negativity and low warmth) reflect an environment that hinders a child’s ability to self-regulate, which may lead to the development of psychopathology^40^. To assess EE, researchers often use the Five-Minute Speech Sample (FMSS;^41)^ and the Expressed Emotion Coding System (EECS;^42)^.

Negative attributions and cognitions in a parent, as seen in those with anxiety and depression^43^, are thought to influence the way in which parents talk about their child through an ‘attribution-negative affect link’^44,45^. Hence, parents with elevated levels of anxiety or depression often display higher levels of EE^45^. Meta-analyses report associations between EE, maternal depression and anxiety, and the severity of children’s internalizing and externalizing behaviors^45,46^. This has led researchers to propose that EE is a transdiagnostic risk factor for the development of internalizing and externalizing problems in children [46,47]. Reviews of the EE literature highlight the need for more comprehensive assessments of the parental emotional environment in order to better understand current findings^46^. Examining both verbal and nonverbal parental emotional communication is one way of providing a more complete understanding of the quality of the child’s emotional environment and its potential influence on children’s internalizing and externalizing problems.

## Current Study

The present study used a novel, multimodal approach to examine associations between maternal emotional context—indexed by maternal depressive and anxious symptoms, verbal expressed emotion (EE), and facial expressivity—and children’s internalizing and externalizing problems. Although maternal anxiety and depression are often comorbid, they represent distinct psychopathologies with potentially differential effects on emotion communication and socialization. Therefore, symptoms of anxiety and depression were analyzed separately rather than combined into a single score. It was hypothesized (1) that higher levels of maternal anxiety and depression would be associated with increased EE, a more maladaptive profile of maternal facial expressivity (i.e., overly negative, diminished, or emotionally discordant), and greater severity of children’s internalizing and externalizing problems; and (2) in multivariate analysis, higher levels of EE and/or maladaptive maternal facial expressivity would be associated with greater severity of children’s internalizing and/or externalizing problems, over and above the effects of maternal anxiety and depression,

## Method

### Participants

The sample consisted of 134 children (31.3% female, 68.7% male) aged 4–8 years old (*M* = 6.46, *SD* = 0.87) and their mothers. The children were referred to the Neurodevelopment Assessment Unit (NDAU; https://www.cardiff.ac.uk/neurodevelopment-assessment-unit) by their teachers because they exhibited emotional, cognitive, and/or behavioral problems at school. Teachers are able to refer a pupil to the NDAU if the child exhibits socio-emotional, cognitive or behavioral difficulties at school, as confirmed by the teacher’s Strengths and Difficulties Questionnaire (SDQ;^48)^ scores. For a child to be accepted for an assessment, they must have an elevated teacher-rated SDQ score in at least one domain of functioning. Although children presented with a range of difficulties, no child had a neurodevelopmental diagnosis at the time of assessment, because having a clinical diagnosis was an exclusion criterion for NDAU referral. Only participants with complete data for the variables of interest were included in the present sample. There were no other inclusion or exclusion criteria.

### Sample Characteristics

Mothers provided child and family background information. This included ethnicity, household income, and parental education, summaries of which are shown in Table 1. Most children were White British (84.3%). Socio-economic status (SES) was assessed using the Welsh Index of Multiple Deprivation (WIMD;^49)^, which is a ranked measure of relative deprivation (where lower numbers indicate greater deprivation). This score takes into consideration several factors, including income, employment, health, and education. According to the WIMD, around a quarter (27.61%) of the sample were living in the most deprived areas of Wales.

**Table 1:** An overview of the sample’s ethnicity, WIMD scores, and maternal education.

	*N*	%
**Child Ethnicity**		
White British	113	84.33%
British/European	6	4.48%
African	3	2.24%
Bangladeshi/Indian/Pakistani	3	2.24%
British/Caribbean	2	1.50%
British/African	2	1.50%
British/Turkish	2	1.50%
Caribbean	1	0.75%
British Bangladeshi/Indian/Pakistani	1	0.75%
British/Arabian	1	0.75%
**WIMD**		
1 (most deprived)	37	27.61%
2	17	12.69%
3	12	8.96%
4	26	19.40%
5 (least deprived)	42	31.34%
**Maternal Education**		
No formal qualification	17	12.69%
O-Levels or GCSEs	39	29.10%
A-Levels/Higher	24	17.91%
Undergraduate degree	28	20.90%
Higher or postgraduate degree	26	19.40%

### Procedure

Mother-child dyads visited the NDAU twice for a neurodevelopmental assessment. Written informed consent was obtained from the primary caregiver for each child prior to the assessment and all experimental procedures were approved by the relevant university (Cardiff University) ethics committee (EC.16.10.11.4592GR). All methods were performed in accordance with the relevant guidelines and regulations. Children completed a range of cognitive, emotional and social assessments, while mothers completed questionnaires and the FMSS in a separate room.

## Measures

### Parent and Child Mental Health

#### Child Internalizing and Externalizing Problems

Symptoms of child internalizing and externalizing behaviors were assessed using the Child Behavior Checklist (CBCL;^50)^. The current study used both the preschool (1.5–5) and child (6–18) versions to accommodate the age range of the sample. T-scores, standardized based on child age and sex, were used as dimensional measures of symptom severity, where scores < 60 are classified as ‘normal’, scores between 60 and 64 are ‘borderline clinical’, and scores > 65 are clinically relevant. These cut-offs were used to describe the sample. The subscales relevant to the current study were the measures of internalizing problems (*α =* 0.872) and externalizing problems (*α =* 0.923).

#### Parent Anxiety and Depression

The Hospital Anxiety and Depression Scale (HADS;^51)^ is a 14-item self-report questionnaire. Seven items measure anxiety symptoms (*α =* 0.796), and 7 items measure depressive symptoms (*α =* 0.794). Items are scored on a 4-point severity scale (0–3). Subscale scores can range from 0 to 21, where 0–7 is seen as normal, 8–10 is borderline abnormal, and scores of 11 or more are clinically relevant.

### Five Minute Speech Sample

#### Expressed Emotion Coding Scheme

The Five-Minute Speech Sample (FMSS;^41)^ is an indirect narrative tool in which the parent is asked to speak freely about their child for 5 min. Each FMSS was recorded, transcribed, and coded using the Expressed Emotion coding scheme^42^. The content and tone of speech are considered to assess the level of warmth and negativity on a global scale of 0–5 (0 reflecting no warmth/negativity), as well as a count of positive and negative comments made during the five minutes. In the current study 57.5% of the sample was coded by the lead coder, and all 5 coders showed good levels of agreement for negative comments (*α =* 0.790), positive comments (*α =* 0.881), and global ratings of warmth (*α =* 0.791) and negativity (*α =* 0.790). The measure has good test-retest reliability and stability over time^52^.

#### Automated Facial Expression Analysis

Facial expressions of mothers were recorded with a Toshiba laptop computer (Satellite Pro-R50-B-123) integrated webcam during the FMSS. Webcam footage was then imported into the iMotions Biometric Research Platform 10.0 software and processed using the Affective AFFDEX facial expression recognition engine. AFFDEX provides scores for 20 ‘channels’ based on the facial action units (FAUs). FAUs used in the current study are shown in *Table **S1* in the *Supplementary Materials*.

This software identifies landmarks on the face (e.g., eyes, chin, and mouth) and assesses the movement and shape of muscle groups responsible for facial expressivity. The output reflects the intensity of FAU activation at pixel level, allowing for very small changes in the intensity of expressivity to be detected. The capacity of the software to detect FAU activation has been reported to be in line with EMG studies^53^. Raw sensor data were used to identify facial behavior during each epoch of interest. Mother’s facial expressivity was recorded throughout the five-minute FMSS task; the epochs of interest were those in which mothers were making positive or negative comments.

### Data Analysis

All analyses were run in IBM SPSS Statistics (version 29.0). All assumptions of normality and multicollinearity were checked prior to analysis. The role of covariates was explored, t-tests were used to check for sex differences, while bivariate correlations were used to examine dimensional covariates (i.e., age and SES). Factor analysis was used to reduce the number of FMSS variables and obtain an overall measure of verbal EE, and to reduce the number of facial expressivity variables. Bivariate correlations were computed to examine potential associations between maternal anxiety, maternal depression, verbal EE, maternal facial expressivity, and children’s internalizing and externalizing problems.

Finally, two hierarchical linear regression models were conducted: one predicting child internalizing problems and another predicting child externalizing problems. In each model, maternal anxiety and depression were entered in the first step, followed by EE in the second step, and maternal facial expressivity in the third and final step to assess whether facial expressivity contributed uniquely to child problems over and above maternal mental health and EE.

## Results

### Preliminary Analyses

Summary statistics relating to sample characteristics are reported in Table 2. There were no significant differences in age, SES, FMSS variables or CBCL scores as a function of sex of child (see *Supplementary Materials*). Summary statistics relating to maternal facial behaviors while making positive and negative comments during the FMSS are shown in *Table S2* (*Supplementary Materials*).

**Table 2:** An overview of the means, standard deviations and score range for FMSS, CBCL and HADS variables, in the total sample, and by child sex.

	Range	Total (*N* = 134)		Male (*N* = 92)		Female (*N* = 42)	
		Mean	SD	Mean	SD	Mean	SD
Age	[4.75, 8.17]	6.46	0.87	6.43	0.83	6.54	0.96
WIMD	[1.00, 5.00]	3.14	1.64	3.13	1.69	3.17	1.55
**FMSS**							
Negative Comments	[0.00, 10.00]	2.62	2.04	2.57	2.08	2.74	1.98
Positive Comments	[0.00, 16.00]	3.62	2.29	3.58	2.20	3.71	2.50
Warmth Rating	[0.00, 5.00]	3.07	1.28	3.11	1.29	2.98	1.28
Negativity Rating	[0.00, 5.00]	1.78	1.12	1.72	1.09	1.93	1.18
**CBCL**							
Internalizing Behaviors	[33.00, 90.00]	63.83	10.60	63.33	10.07	64.97	11.81
*Borderline (%)*		*10.71%*		*10.26%*		*11.76%*	
*Clinical (%)*		*55.36%*		*52.56%*		*58.82%*	
Externalizing Behaviors	[44.00, 92.00]	67.90	10.68	67.81	11.02	68.12	10.00
*Borderline (%)*		*8.04%*		*6.41%*		*11.76%*	
*Clinical (%)*		*69.79%*		*69.23%*		*70.59%*	
Total Problem Behaviors	[46.00, 87.00]	69.25	8.48	68.90	8.61	70.06	8.22
*Borderline (%)*		*8.93%*		*12.82%*		*0.00%*	
*Clinical (%)*		*75.00%*		*70.51%*		*85.29%*	
**HADS**							
Anxiety Symptoms	[1.00, 19.00]	8.49	4.05				
*Borderline (%)*		*30.08%*					
*Clinical (%)*		*28.46%*					
Depression Symptoms	[0.00, 19.00]	5.71	3.92				
*Borderline (%)*		*12.20%*					
*Clinical (%)*		*14.63%*					

Note. Abbreviations: **CBCL**, Child Behavior Checklist; **FMSS**, Five Minute Speech Sample; **HADS**, Hospital Anxiety and Depression Scale.

### Data reduction

These four variables were highly correlated with one another (see *Table S3*,* Supplementary Materials*), and the Kaiser-Meyer-Olkin value and Bartlett test of sphericity confirmed that structure detection was appropriate for these FMSS variables (KMO = 0.627; χ2 (6) = 240.54, *p* < .001), in line with previous research^47^. To understand the underlying relationships between these variables, Direct Oblimin rotation was used, and the rotated factor loadings are shown in Table 3. All variables loaded highly onto a single factor, labelled ‘EE_Neg_’, reflecting the fact that negativity and negative comments had high positive loadings whereas positive comments and warmth had high negative loadings. This factor explained 65.52% of the variance in FMSS scores.

**Table 3:** Factor loadings and communalities for Direct Oblimin rotated one-factor solution for 4 FMSS variables.

	Factor loading	Communality
Negativity Rating	0.872	0.761
Negative Comments	0.789	0.623
Positive Comments	− 0.598	0.357
Warmth Rating	− 0.871	0.759

To reduce the number of FAU activation measures, FAUs during negative and positive comments, were entered into a PCA. Varimax rotation resulted in a five-factor solution, as shown in Table 4, KMO = 0.577; χ2 (105) = 1112.82, *p* < .001. Together these factors explained 76.06% of the variance in FAU activation scores. Factor 1 included high loadings for ‘Cheek Raise’ and ‘Smile’, and moderate loadings for ‘Lid Tighten’ across both positive and negative comments and was interpreted as reflecting a ‘Duchenne Smile’, a genuine smile involving *Orbicularis Oculi* and *Zygomatic Major* activation^37^. Factor 2 included high loadings for ‘Brow Furrow’ and ‘Inner Brow Raise’ during both positive and negative comments and was labelled ‘Brow Expressivity’. Factor 3 had high loadings for ‘Brow Furrow’, ‘Lid Tighten’ and ‘Nose Wrinkle’ and was interpreted as expressing anger, but only during positive comments, and was therefore labelled ‘Anger (P)’. Factor 4 had high loadings for ‘Lip Corner Depressor’ during both positive and negative comments and was simply labelled Lip Corner Depressor. Finally, Factor 5 had high loadings for ‘Brow Furrow’, and ‘Lid Tighten’ and moderate loadings for ‘Nose Wrinkle’, but only during negative comments, and was labelled ‘Anger (N)’.

**Table 4:** PCA of FAU activation scores during negative and positive comments.

		1. Duchenne smile	2. Brow expressivity	3. Anger (*P*)	4. Lip corner depressor	5. Anger (*N*)
Smile	0.884	-	-	-	-	
Nose wrinkle	-	-	0.484	-	0.494	
Lid tighten	0.468	-	-	-	0.720	
Lip corner dep	-	-	-	0.894	-	
Inner brow raise	-	0.779	-	-	-	
Cheek raise	0.897	-	-	-	-	
Brow raise	-	0.792	-	-	-	
Negativity rating	Brow Furrow	-	-	-	-	0.814
Positive comments	Brow Furrow	-	-	0.705	-	-
	Brow Raise	-	0.750	-	-	-
	Cheek Raise	0.883	-	-	-	-
	Inner Brow Raise	-	0.751	-	-	-
	Lip Corner Dep	-	-	-	0.875	-
	Lid Tighten	0.445	-	0.760	-	-
	Nose Wrinkle	-	-	0.740	-	-
	Smile	0.879	-	-	-	-
Explained variance (%)		24.31	14.94	13.83	11.90	11.07
Eigenvalue		3.89	2.39	2.21	1.90	1.77

Note. Coefficients < 0.4 are not shown.

Correlations were conducted between these maternal facial expressivity factors, maternal emotional environment (EE_Neg_ and HADS scores), child internalizing and externalizing problems, with age and SES as covariates (sex was excluded due to nonsignificant differences). As summarized in Table 5, child age was associated with more maternal brow expressivity and more lip corner depressor movement. Maternal anxiety and depression were significantly positively correlated with one another, and with child internalizing and externalizing problems. Maternal depression was associated with increased EE_Neg_, which was in turn associated with increased internalizing and externalizing problems. More deprivation (WIMD) was associated with less anger when talking positively about the child.

Correlation coefficients also revealed interesting associations between maternal expressivity and internalizing and externalizing problems. Although fewer and less intense maternal facial expressions tended to be associated with more internalizing and externalizing problems, the only significant correlation was one showing that less intense and less frequent expressions of facial anger during instances of negative comments were associated with more severe internalizing problems in children.

**Table 5:** Correlations Among, HADS, EE, CBCL, and Maternal Facial Expressivity Scores.

	1	2	3	4	5	6	7	8	9	10	11	12
1. Age	--											
2. WIMD	0.036	--										
3. HADS anxiety	0.041	0.016	--									
4. HADS depression	0.071	− 0.142	0.641^**^	--								
5. EE_Neg_	0.157	− 0.058	0.155	0.232^**^	--							
6. CBCL internalizing	− 0.095	− 0.145	0.301^**^	0.262^**^	0.198^*^	--						
7. CBCL externalizing	− 0.036	− 0.114	0.243^*^	0.272^**^	0.517^**^	0.513^**^	--					
8. Duchenne smile	− 0.123	− 0.014	− 0.050	− 0.049	− 0.156	− 0.049	− 0.114	--				
9. Brow expressivity	0.239^*^	− 0.099	− 0.112	− 0.109	0.128	− 0.206	− 0.011	0.025	--			
10. Anger (P)	0.035	0.243^*^	− 0.048	− 0.207^*^	− 0.274^**^	− 0.092	− 0.079	− 0.079	0.005	--		
11. Lip corner depressor	0.199^*^	0.084	− 0.037	− 0.134	− 0.021	− 0.143	− 0.129	0.151	0.164	− 0.029	--	
12. Anger (N)	0.144	− 0.026	− 0.086	0.038	0.125	− 0.331^**^	− 0.141	− 0.155	0.068	− 0.112	0.186	--

* Correlation is significant at the 0.05 level (2-tailed).

** Correlation is significant at the 0.01 level (2-tailed).

Children’s internalizing problems were regressed on maternal anxiety and depression (Step 1), EE (Step 2), and facial expressivity (Step 3). The results are summarized in Table 6. The overall model for Step 1 was statistically significant (*F*(2, 82) = 4.69, *p* = .012), accounting for 10.3% of the variability in internalizing scores. Maternal anxiety was the only significant predictor (*β* = 0.306, *p* = .040). The addition of EE_Neg_ in Step 2 resulted in a non-significant change, showing that EE_Neg_ did not add significantly to what was predicted by maternal anxiety. Adding maternal facial expressivity in Step 3 resulted in a significant change, explaining an additional 11.8% of the variance in internalizing scores, *F*(5, 78) = 2.31, *p* = .049. The only significant individual predictor of internalizing problems in Step 3 was Anger (N), *β*= − 0.314, *p* = .004. The negative beta weight shows that mothers who expressed less anger while making negative comments had children with higher internalizing scores.

**Table 6:** Summary of hierarchical regression regressing CBCL Internalizing on to Maternal Anxiety and Depression, EE_neg_, and Maternal Facial Expressivity.

Variable	B	95% CI	β	sr	t	*p*
**Step 1, ∆ R²= 0.103**						
HADS anxiety	0.722	[0.035, 1.41]	0.306	0.219	2.09	0.040
HADS depression	0.047	[-0.647, 0.740]	0.02	0.014	0.134	0.893
**Step 2, ∆ R²= 0.002**						
HADS anxiety	0.726	[0.035, 1.42]	0.308	0.220	2.09	0.040
HADS depression	0.014	[-0.700, 0.730]	0.006	0.004	0.039	0.969
EE_Neg_	0.497	[-1.77, 2.76]	0.048	0.046	0.437	0.663
**Step 3, ∆ R²= 0.118**						
HADS anxiety	0.509	[-0.180, 1.20]	0.216	0.149	1.47	0.145
HADS depression	0.026	[-0.680, 0.733]	0.011	0.008	0.074	0.941
EE_Neg_	0.895	[-1.35, 3.14]	0.086	0.080	0.793	0.430
Duchenne smile	− 0.305	[-2.20, 1.59]	− 0.033	− 0.032	− 0.32	0.750
Brow expressivity	-1.41	[-3.32, 0.500]	− 0.153	− 0.149	-1.47	0.146
Anger (P)	− 0.638	[-2.39, 1.11]	− 0.075	− 0.073	− 0.726	0.470
Lip corner depressor	− 0.050	[-1.83, 1.73]	− 0.006	− 0.006	− 0.055	0.956
Anger (N)	-3.17	[-5.29, -1.06]	− 0.314	− 0.302	-2.99	0.004

Note. R^2^_adj_ = 0.141 (*N* = 134, *p* = .049). CI= Confidence Interval for *B*,* sr* = semipartial correlation coefficient.

In a parallel analysis of children’s externalizing problems (summarized in Table 7), the model was significant at Step 1, *F*(2, 82) = 4.69, *p* = .010, accounting for 10.7% of the variance in externalizing scores. Maternal depression was the only significant predictor (*β* = 0.348, *p* = .020). Adding EE_Neg_ (*β* = 0.398, *p*= .000) in Step 2 resulted in a significant change, *F*(1, 83) = 15.93, *p* < .001, explaining an additional 14.7% of the variance. Adding maternal facial expressivity in Step 3 did not result in a significant change, despite accounting for an additional 5.5% variance (*F*(5, 78) = 1.21, *p* = .315). However, Anger (N) was a significant individual predictor, *β* = − 0.205, *p* < .05, together with EE_Neg_, *β* = 0.415, *p* < .001. The beta weights associated with Anger (N) and EE_Neg_ show that mothers who expressed more EE_Neg_ and mothers who expressed less anger while making negative comments had children with higher externalizing scores.

**Table 7:** Summary of hierarchical regression regressing CBCL Externalizing on to Maternal Anxiety and Depression, EE_neg_, and Maternal Facial Expressivity.

Variable	B	95% CI	β	sr	t	*p*
**Step 1, ∆ R²= 0.107**						
HADS anxiety	− 0.077	[-0.802, 0.647]	− 0.031	− 0.022	− 0.212	0.833
HADS depression	0.876	[0.145, 1.61]	0.348	0.249	2.38	0.020
**Step 2, ∆ R²= 0.147**						
HADS anxiety	− 0.048	[-0.714, 0.619]	− 0.019	− 0.014	− 0.142	0.887
HADS depression	0.586	[-0.102, 1.27]	0.233	0.163	1.70	0.094
EE_Neg_	4.38	[2.20, 6.56]	0.398	0.383	3.99	0.000
**Step 3, ∆ R²= 0.055**						
HADS anxiety	− 0.168	[-0.855, 0.520]	− 0.067	− 0.046	− 0.486	0.629
HADS depression	0.591	[-0.114, 1.30]	0.235	0.159	1.67	0.099
EE_Neg_	4.57	[2.32, 6.81]	0.415	0.387	4.06	0.000
Duchenne smile	− 0.527	[-2.42, 1.37]	− 0.055	− 0.053	− 0.554	0.581
Brow expressivity	− 0.354	[-2.26, 1.55]	− 0.036	− 0.035	− 0.370	0.712
Anger (P)	− 0.771	[-2.52, 0.980]	− 0.086	− 0.084	− 0.879	0.382
Lip corner depressor	0.188	[-1.59, 1.97]	0.020	0.020	0.210	0.834
Anger (N)	-2.19	[-4.31, − 0.079]	− 0.205	− 0.197	-2.066	0.042

Note. R^2^_adj_ = 0.236 (*N* = 134, *p* = .315). CI= Confidence Interval for *B*,* sr* = semipartial correlation coefficient.

## Discussion

Mothers are seen as the primary context for a child’s emotional development^5,7^. The present study aimed to improve our understanding of how different aspects of the maternal emotional environment are associated with a child’s internalizing and externalizing problems, with a specific focus on examining the added value of measuring mothers’ facial expressivity. Results confirm the importance of taking a multimodal approach to understanding maternal influences on a child’s emotional and behavior problems and indicate that mothers’ verbal and nonverbal emotional expressions play a role in the severity of internalizing and externalizing problems in young children.

Maternal anxiety and depression symptoms have been associated with maladaptive outcomes for children^54,55^. In the current study, the experience of borderline or clinically relevant symptoms of anxiety (58.54%) and depression (26.83%) was relatively higher than global norms^56^ and demonstrated that maternal anxiety and depression are highly comorbid^57^. Consistent with findings that mothers with anxiety are more likely to have children with anxiety or other internalizing problems^55^, regression analysis indicated that maternal anxiety predicted the severity of child internalizing problems over and above maternal depression. Maternal depression, however, is thought to predispose children to an increased risk of developing any psychopathology^54^. In line with this, we found that although correlations showed that maternal depression was associated with both internalizing and externalizing problems in children, only maternal depression predicted the severity of externalizing problems in regression models.

In line with previous research, an EE factor was constructed^47^. This factor encompasses EE as a concept, considering both warmth and negativity. This allows for a more accurate understanding of how EE relates to parent mental health and child internalizing and externalizing behaviors, as the factor is more reflective of how warmth and negativity interact with one another in a ‘real-world’ context. In the current study, correlations indicated that EE was associated with increased severity of maternal depression, as in previous research^44,45^ but was not related to the severity of maternal anxiety. Attributional models of EE support this finding, suggesting that individuals with propensities to negative attributions – often seen in depression – make more negative comments about their child^44,45^. It is important to note, however, that correlations should be interpreted with caution, given that they do not control for other variables or adjust for multiple comparisons. Correlations also indicated that EE was associated with severity of child internalizing and externalizing problems^45,46^, but importantly, in regression analyses, EE was a better predictor of externalizing problems than parental depression and/or anxiety. This pattern of results is in line with proposals that EE is a ‘child-specific’ environmental influence^42^ and that persistent stress in the form of parental negativity impacts the child’s ability to regulate their emotions, which can result in the development of externalizing problems^40^.

Regression analyses showed that fewer and less intense expressions of maternal anger during instances of negative comments were a significant predictor of child internalizing problems, whereas a combination of negative verbally expressed emotion (EE) and fewer and less intense facial expressions of anger during negative statements about the child significantly predicted the severity of externalizing problems in young children. In the FMSS, the focus of the negativity coding is on verbal expressions of anger and dissatisfaction with the child or their behaviors^42^ and one would therefore expect facial expressivity to reflect mild or moderate anger (i.e., furrowed brow, tightened eyes, nose wrinkle;^37)^. Therefore, fewer and less intense expressions of anger during negative comments could reflect instances in which mothers are not modelling the expected emotion. Adaptive expression and congruent emotion related behaviors, such as talking about emotions and reacting to emotions appropriately, ensure that children acquire critical emotional skills in early life^58^. This discordance between maternal facial affect and the content of their speech could, in an interaction with a child, negatively impact a child’s emotion socialization^5^, through maladaptive emotional teaching, creating a lack of emotional security for the child^18^. Maladaptive emotional processes learnt through modelling maternal emotion socialization processes are further associated with internalizing and externalizing behaviors in young children^59,60^. Exacerbated by their emotion recognition difficulties^60^, children with externalizing problems may struggle to understand discordant emotional cues and therefore fail to understand the consequences of their actions, continuing to display aggressive or oppositional behaviors.

Previous research using self-reports or observational data from parent-child interactions to measure maternal facial affect reported that increases in negative facial affect or decreases in positive affect are associated with more severe internalizing and externalizing problems in children^13,15,16^. A strength of the current study is the assessment of maternal facial expressivity outside their interactions with their child. Self-report measures of one’s own facial expressivity are known to be inaccurate^28,29^ and parents’ facial expressions are generally more positive and less negative in direct interactions with a child^15^. This is likely a result of social desirability biases or observer effects^61^. Hence, the assessment of maternal facial behavior implemented in the current study is thought to reflect trait expressivity, meaning it is more representative of what a child would experience in the home. This novel approach allowed for the accurate assessment of more specific facial behaviors in positive and negative contexts, leading to the conclusion that a lack of *anger* expression when mothers are talking negatively about their child is associated with increased internalizing and externalizing behaviors.

Although few correlations between maternal facial expressivity and child outcomes were statistically significant, adding maternal facial expressivity as a predictor in regression models added significantly to the explanation of the severity of child’s internalizing and externalizing problems, beyond maternal mental health and EE. This highlights the value of facial expressivity to multimodal models of emotion socialization and shows that inclusion of nonverbal emotional indicators can help to uncover subtleties in emotion modelling that would otherwise be overlooked. While AFEA may be of limited use in clinical contexts due to the complexity of its measurement^38^, when it comes to theoretical models of emotional development in children AFEA can help to detect meaningful patterns of emotional communication that verbal measures alone cannot capture^5–7^. In particular, the current research reveals inconsistencies in maternal verbal and nonverbal expressivity that could affect a young child’s ability to decode and interpret emotional signals^18^.

As with many developmental models of emotion socialization, a child’s emotional and behavioral adjustment are considered the ‘outcome’^5–7^. However, our study did not have a longitudinal design, and it is important to recognize that children are active agents in their own development, with bidirectional effects being most likely^4,62^. Aversion sensitivity models suggest that parents, particularly those who experience mood disorders, may struggle to regulate their emotions, and may modify or suppress facial expressions to avoid escalating child distress^27,63^. Although a reduction in facial expressivity might reduce immediate child distress, it may also limit opportunities for children to learn about emotion regulation and appropriate emotional responses^5^. This may contribute to maladaptive emotion socialization, whereby children learn that emotions are threatening and should be avoided, reducing their ability to understand and respond adaptively to their own emotions and those of others^5–7^. Within the context of the present study, mothers of children with more severe internalizing and externalizing problems were found to express fewer and less intense expressions of anger while speaking negatively about their child. This pattern may reflect child-elicited effects, whereby challenging child behaviors lead mothers to suppress or modulate their anger, consistent with aversion sensitivity processes, highlighting the reciprocal nature of parent–child emotional dynamics^27,63^.

### Limitations

The current study was cross-sectional, and therefore cannot establish how these problems emerge, or definitively conclude that variation in maternal depression and anxiety, EE or facial expression precedes the development of internalizing or externalizing problems in children. Developmental models of emotion socialization propose that the parent is the primary source of emotional information and construct the emotional environment in which a child develops^5^. This is not to say, however, that the children are not active agents in their own development. Parent–child interactions are transactional, and child temperament, behavior and personality are all known to influence the way that parents act towards their child^4,62^. Measures of the maternal emotional environment are thought to be stable over time^52^, but the use of transactional or longitudinal models in future research would further aid the understanding of these issues.

Additionally, there are limitations related to the sample that should be considered when interpreting the results. Children were referred for an assessment due to emotional, cognitive and/or behavioral problems at school, so the findings reflect associations between parents and children that may not reflect what is seen in community samples^64^. While high-risk samples can provide valuable insight into how risk is transmitted in family contexts, these effects may not be observed in typical samples, where patterns may instead reflect the promotion of resilience through adaptive parental expressivity^8,9,13,14^. Furthermore, the majority of participants identified as White British, which limits the generalizability of the findings to more diverse populations. Cultural factors are known to shape parenting practices, emotional expressions, and parent–child interactions^65^, suggesting that both the methods and observed associations could differ in families from other cultural or ethnic backgrounds. Future research should aim to replicate these findings in more diverse samples.

Our study was the first to use AFEA in a relatively large sample of at-risk mothers and children, focusing on the role of maternal verbal and facial expressivity. While AFEA provides an objective measure of facial behavior^34^, a caveat is that it does not consider the wider emotional context of the situation, such as bodily or vocal expressions of emotion. Because AFEA relies on algorithmic identification of facial behavior, it may overlook information that a human observer would deem important. Hence, an even broader multimodal approach, combining AFEA with observer or self-report measures of expressivity might offer a more robust understanding of the experience and expression in emotion socialization models.

Future research in high-risk samples of mothers and children should also aim to examine the direct effects of maternal mood states and verbal and facial emotion expressions on the child. Instead of focusing on child clinical symptoms, studies could instead assess children’s emotion recognition, their affective and cognitive empathy, and other forms of prosocial behavior, and incorporate, if possible, measures of physiological responsivity^5^.

## Conclusion

The results of the current study confirm the importance of considering a wider perspective of the maternal emotional environment on the severity of a child’s internalizing and externalizing behavior problems. Results suggest that less intense maternal negative facial affect when talking about their child in a negative way is associated with more severe internalizing and externalizing problems in the child. When children are exposed to inconsistent emotional signals, they may fail to develop an adequate understanding of others’ emotions and of how to respond appropriately. This understanding is also essential for emotion-based and parenting interventions that are designed to support children’s prosocial development.

## Supplementary Information

Below is the link to the electronic supplementary material.

Supplementary Material 1

## Data Availability

The conditions of our ethics approval do not permit public archiving of anonymized study data. Access to the data can be requested by contacting the lead investigator (SvG) or the local ethics committee at the School of Psychology, Cardiff University. Access will be granted to named individuals in accordance with ethical procedures governing the re-use of sensitive data. Specifically, requestors must complete a formal data sharing agreement with the lead investigator.
