# Supplementary material for: Beyond Words: Associations between the Maternal Emotional Environment and Children’s Internalizing and Externalizing Symptoms
Source: Sci Rep. 2026 Apr 13;16:17200. doi: 10.1038/s41598-026-47543-1 (PMC13234360; doi:10.1038/s41598-026-47543-1)
Supplement: Supplementary file 1 — Supplementary Material 1 [file 41598_2026_47543_MOESM1_ESM.pdf]

Supplementary Materials

Beyond Words: Associations between the Maternal Emotional Environment and Children's Internalizing and Externalizing Symptoms, Scientific Reports

Table S1. Description facial action units measured in the current study

Facial Action Unit	Description	Facial Muscle
1	Inner Brow Raise	*Frontalis, pars medialis*
2	Brow Raise	*Frontalis, pars lateralis*
4	Brow Furrow	*Depressor glabellae, depressor supercilli, currugator*
6	Cheek Raise	*Orbicularis oculi, pars orbitalis*
7	Lid Tighten	*Orbicularis oculi, pars palpebralis*
9	Nose Wrinkle	*Levator labii superioris alaeue nasi*
12	Lip Corner Puller (Smile)	*Zygomatic Major*
15	Lip Corner Depressor	*Depressor anguli oris (Triangularis)*

Table S2. Ranges, means, and standard deviations for FAU variables

FAU	Negative Comments			Positive Comments		
	Range	Mean	SD	Range	Mean	SD
Brow Furrow	[0.00, 47.48]	4.08	7.36	[0.00, 44.83]	3.39	6.11
Brow Raise	[0.04, 85.09]	14.72	18.61	[0.11, 80.33]	18.15	19.68
Cheek Raise	[0.17, 61.81]	11.04	14.19	[0.19, 93.76]	16.08	17.90
Inner Brow Raise	[0.02, 51.06]	4.38	7.43	[0.00, 53.52]	6.35	9.66
Lip Corner Depressor	[0.00, 81.22]	3.34	8.65	[0.00, 44.39]	2.89	6.14
Lid Tighten	[0.01, 56.40]	7.35	10.13	[0.01, 51.00]	7.81	10.30
Nose Wrinkle	[0.03, 49.97]	6.23	7.63	[0.04, 38.10]	5.98	7.22

Smile	[0.07, 71.89]	15.73	18.33	[0.10, 99.93]	23.27	23.95

Table S3. Pearson Correlations between FMSS variables

	1	2	3	4
1. Negative Comments	-			
2. Positive Comments	-.121	-		
3. Warmth Rating	-.533**	.596**	-	
4. Negativity Rating	.742**	-.323**	-.608**	-

**. Correlation is significant at the 0.01 level (2-tailed).

*. Correlation is significant at the 0.05 level (2-tailed).

Independent-samples *t* tests

- **Age:** No significant group difference, $t(132) = -0.82$, $p = .415$, 95% CI [−5.81, 2.41].
- **WIMD quintile:** No significant group difference, $t(132) = -0.12$, $p = .906$, 95% CI [−0.64, 0.57].
- **FMSS negative comments:** No significant group difference, $t(132) = -0.45$, $p = .651$, 95% CI [−0.93, 0.58].
- **FMSS positive comments:** No significant group difference, $t(132) = -0.32$, $p = .747$, 95% CI [−0.98, 0.71].
- **FMSS warmth:** No significant group difference, $t(132) = 0.55$, $p = .581$, 95% CI [−0.34, 0.61].
- **FMSS negativity:** No significant group difference, $t(132) = -1.01$, $p = .313$, 95% CI [−0.62, 0.20].
- **CBCL internalizing problems:** No significant group difference, $t(110) = -0.75$, $p = .455$, 95% CI [−5.96, 2.69].
- **CBCL externalizing problems:** No significant group difference, $t(110) = -0.14$, $p = .888$, 95% CI [−4.68, 4.06].
- **HADS anxiety:** No significant group difference, $t(121) = 0.12$, $p = .906$, 95% CI [−1.46, 1.65].
- **HADS depression:** No significant group difference, $t(121) = 0.01$, $p = .989$, 95% CI [−1.49, 1.51].
